# Nomogram for predicting preoperative lymph node involvement in patients with invasive micropapillary carcinoma of breast: a SEER population-based study

**DOI:** 10.1186/s12885-018-4982-5

**Published:** 2018-11-08

**Authors:** Fu-Gui Ye, Chen Xia, Ding Ma, Pei-Yang Lin, Xin Hu, Zhi-Ming Shao

**Affiliations:** 1 Key Laboratory of Breast Cancer in Shanghai, Department of Breast Surgery, Fudan University Shanghai Cancer Center, Shanghai, China; 2 Department of Oncology, Shanghai Medical College, Fudan University, Shanghai, China; 3 The Affiliated Cancer Hospital of Xiangya School of Medicine, Central South University, Changsha, China; 4 Department of Breast Surgery, Affiliated Union Hospital, Fujian Medical University, Fuzhou, China; 5 Institutes of Biomedical Science, Fudan University, Shanghai, China

**Keywords:** IMPC, Breast cancer, Preoperative, Predict, Lymph nodes involvement

## Abstract

**Background:**

Invasive micropapillary carcinoma (IMPC) is an unusual and distinct subtype of invasive breast tumor with high propensity for regional lymph node metastases. This study was to identify risk factors accounting for IMPC of the breast and to develop a nomogram to preoperatively predict the probability of lymph node involvement.

**Methods:**

A retrospective review of the clinical and pathology records was performed in patients diagnosed with IMPC between 2003 and 2014 from Surveillance, Epidemiology, and End Results (SEER) database. The cohort was divided into training and validation sets. Training set comprised patients diagnosed between 2003 and 2009, while validation set included patients diagnosed thereafter. A logistic regression model was used to construct the nomogram in the training set and then varified in the validation set. Nomogram performance was quantified with respect to discrimination and calibration using R 3.4.1 software.

**Results:**

Overall, 1407 patients diagnosed with IMPC were enrolled, of which 527 in training set and 880 in validation set. Logistic regression analysis indicated larger lesions, younger age at diagnosis, black ethnic and lack of hormone receptor expression were significantly related to regional nodes involvement. The AUC of the nomogram was 0.735 (95% confidential interval (CI) 0.692 to 0.777), demonstrating a good prediction performance. Calibration curve for the nomogram was plotted and the slope was close to 1, which demonstrated excellent calibration of the nomogram. The performance of the nomogram was further validated in the validation set, with AUC of 0.748 (95% CI 0.701 to 0.767).

**Conclusions:**

The striking difference between IMPC and IDC remains the increased lymph node involvement in IMPC and therefore merits aggressive treatment. The nomogram based on the clinicalpathologic parameters was established, which could accurately preoperatively predict regional lymph node status. This nomogram would facilitate evaluating lymph node state preoperatively and thus treatment decision-making of individual patients.

## Background

Invasive micropapillary carcinoma (IMPC) of the breast was first described by Fisher et al. in 1980 [1], and then defined by Siriaunkgul and Tavassoli in1993 [2]. In 2003 World Health Organization (WHO) guidelines for histologic classification of tumors of the breast, IMPC was considered as a rare subtype of invasive breast carcinoma, accounting for approximately 2% to 8% of all breast cancers [3]. Over the past decades, a series of studies have conducted to explore clinical-pathologic characteristics, clinical outcomes, prognostic factors and underlying mechanisms of IMPC.

Fundamental research concerning IMPC, in conjunction with clinical practice, had confirmed that IMPC was linked to high frequency of lymph nodal metastasis (LNM) and lymphovascular invasion (LVI) [4–8]. Axillary lymph node metastasis is one of the most important prognostic determinants for patients with breast cancer. Accurately preoperative assessment of lymph node involvement has become an essential issue with respect to determining the need for neoadjuvant therapy and aiding in axillary lymph nodes dissection decision making or other alternative treatment options.

However, the relatively low incidence of IMPC makes it robustly difficult to characterize the nature course of this aggressive subset of breast carcinoma. Till now, the factors contributing to the lymphotropic nature of IMPC had not yet been fully understood and need to be further elucidated. Although studies indicated that tumor-infiltrating lymphocytes (TILs) [9], cytokines, membrane proteins (such as stromal cell-derived factor-1 and its receptor CXCR4, caveolin-1) [10–12], epigenetic regulators (such as miRNAs (let-7b, miR-30c, miR-148a, miR-181a, miR-181b), promoter hypermethylation of the *LZTS1* gene) [13, 14] were associated with lymph node metastasis, there was no convenient nomogram facilitating preoperative prediction of the lymph node involvement.

The objective of this retrospective study was to determine the clinicopathologic characteristics correlating with lymphotropic nature of IMPC. Furthermore, a nomogram was constructed by applying the identified factors to predict the lesions likely to be regional lymph node involvement prior to surgery.

## Methods

### Ethical statement

This study was approved by the Ethical Committee of the Shanghai Cancer Center of Fudan University. The data released from the SEER database did not require informed patient consent because cancer is a reportable disease in every state in the US.

### Patient selection

The SEER*Stat version 8.3.4 was used to generate a case listing. A total of 1407 patients was eligible and enrolled according to the following inclusion criteria: year of diagnosis from 2003 to 2014, pathologically confirmed invasive micropapillary carcinoma of the breast (ICD-O-3 8507), unilateral breast cancer, adjusted AJCC 6th stage, known tumor size, regional lymph nodes involvement, ER and PR status. Patients with no record of regional lymph node status and tumor size and diagnosed before 2003 were excluded from this analysis. Comparable clinicopathologic characteristics of IMPC patients diagnosed between the periods of 2003 to 2009 and 2010 to 2014, therefore, the training set comprised patients diagnosed between 2003 to 2009, while validation set included patients diagnosed thereafter.

### Statistical analysis

Continuous variables were compared between the training set and validation set using Mann-Whitney U tests, and categorical variables were analysed using the Pearson’s chi-square test or Fisher’s exact test when needed. To identify factors that were associated with regional lymph node involvement, binary logistic regression analysis was used for multivariable analysis. Odds ratios (OR) were presented with 95% CI. Preoperatively available variables were included in the logistic regression analysis.

To construct a well calibrated and discriminative nomogram for predicting regional lymph node metastasis, a model was developed in a training set and then validated in another data set. A logistic regression model was used to construct the nomogram. Variables with *P* <0.05 were included in the nomogram, unless otherwise specified. A likelihood ratio test approach for model selection was performed.

Nomogram performance was quantified with respect to discrimination and calibration. Discrimination (the ability of a nomogram to separate patients with different lymph node status) was quantified by means of the area under the receiver operating characteristic (ROC) curve (AUC or C-index). Calibration was assessed graphically by plotting the relationship between actual (observed) probabilities and predicted probabilities (calibration plot) by using Hosmer goodness-of-fit test [15]. Internal validation of performance was estimated with the bootstrapping method (1000 replications). All tests were two-sided, and *P*<0.05 was deemed significant. Statistical analyses were conducted using SPSS for windows (version 22.0, SPSS Inc., Chicago, IL, USA) and the R programming language and environment version 3.4.1 (http://cran.r-project.org).

## Results

### Clinical and pathologic characteristics of the study cohort

The clinicopathologic features of the study cohort were listed in Table 1. The cohort included 1407 patients with a median follow-up time of 37 (25–75%, 15–69) months. The median survival times of the primary and validation cohorts were 79 months (25–75%, 64–97) and 22 months (25–75%, 9–37), respectively. Similar to invasive ductal carcinoma (IDC) of the breast, an overwhelming majority patients were female (98.22%). The median ages at diagnosis of the primary and validation set were 60 (25–75%, 51–70) and 61 (25–75%, 52–70), respectively. The breast cancer lesion was located in the left breast in 724 patients (51.46%) and in the right breast in 683 patients (48.54%).

**Table 1:** Patients’ demographics and clinicalpathologic characteristics

Characteristics	Training Cohort		Validation Cohort		All Patients	
	*N* = 527 (%)		*N* = 880 (%)		*N* = 1407 (%)	
Median follow-up (IQR)	79 (64–97)		22 (9–37)		37 (15–69)	
Median age at diagnosis (IQR)	60 (51–70)		61 (52–70)		61 (51–70)	
Ethnicity						
White	418	79.32	675	76.70	1093	77.68
Black	60	11.39	111	12.61	171	12.15
Others	49	9.30	94	10.68	143	10.16
Gender						
Male	4	0.76	21	2.39	25	1.78
Female	523	99.24	859	97.61	1382	98.22
Laterality						
Left	281	53.32	443	50.34	724	51.46
Right	246	46.68	437	49.66	683	48.54
Tumor size(cm)						
<2	283	53.70	461	52.39	744	52.88
2–5	189	35.86	316	35.91	505	35.89
≥ 5	55	10.44	103	11.70	158	11.23
ER status						
Positive	458	86.91	801	91.02	1259	89.48
Negative	69	13.09	79	8.98	148	10.52
PR status						
Positive	388	73.62	707	80.34	1095	77.83
Negative	139	26.38	173	19.66	312	22.17
HER2 status						
Positive	NA	NA	171	19.43	171	12.15
Negative	NA	NA	679	77.16	679	48.26
Borderline	NA	NA	22	2.50	22	1.56
Unknown	NA	NA	8	0.91	8	0.57
Histological grade						
I	48	9.11	53	6.02	101	7.18
II	247	46.87	499	56.70	746	53.02
III	216	40.99	307	34.89	523	37.17
Unknown	16	3.04	21	2.39	37	2.63
Regional lymph nodes						
Positive	263	49.91	447	50.80	710	50.46
Negative	264	50.09	433	49.20	697	49.54
Stage						
I	196	37.19	328	37.27	524	37.24
II	211	40.04	343	38.98	554	39.37
III	113	21.44	194	22.05	307	21.82
IV	5	0.95	8	0.91	13	0.92
Unknown	2	0.38	7	0.80	9	0.64

*IQR* interquartile range, *ER* estrogen receptor, *PR* progesterone receptor, *HER2* human epidermal growth factor 2

As to the routine immunoprofiles, ER status showed positive rate of 89.48%, and 77.83% for PR status. In the present study, 50.46% (710/1407) of patients were confirmed to pathological lymph node metastasis, with positive rate of 49.91% and 50.80% in the primary and validation cohorts, respectively. There were no significant differences between the primary and validation groups with regard to clinicopathologic characteristics.

### Factors associated with preoperative axillary lymph node metastasis

To identify factors potentially predict axillary lymph node metastasis, univariate analysis was performed in the primary cohort as indicated in Table 2. The results demonstrated that factors most strongly associated with preoperative axillary lymph node involvement were older age at diagnosis, tumor size, histological grade, and stage. Of note, the ER status, which was quantified as one of the most important drivers of breast cancer development, progression and metastasis, showed no significant relation with positive axillary lymph nodes, while PR status had marginal correlation with that.

**Table 2:** Univariable association analyses in the training and validation cohorts

Characteristics	Training Cohort					Validation Cohort				
	LN Metastasis (+)		LN Metastasis (−)		*p*	LN Metastasis (+)		LN Metastasis (−)		*p*
Age at diagnosis	NO.	%	NO.	%	<0.001	NO.	%	NO.	%	0.001
≤ 50	83	31.56	43	16.29		121	27.07	75	17.32	
>50	180	68.44	221	83.71		326	72.93	358	82.68	
Ethnicity					0.129					0.033
White	207	78.71	211	79.92		334	74.72	341	78.75	
Black	36	13.69	24	9.09		69	15.44	42	9.70	
Others	20	7.60	29	10.98		44	9.84	50	11.55	
Gender					0.997					0.018
Male	2	0.76	2	0.76		16	3.58	5	1.15	
Female	261	99.24	262	99.24		431	96.42	428	98.85	
Laterality					0.175					0.502
Left	148	56.27	133	50.38		230	51.45	213	49.19	
Right	115	43.73	131	49.62		217	48.55	220	50.81	
Tumor size(cm)					<0.001					<0.001
<2	98	37.26	185	70.08		142	31.77	319	73.67	
2–5	117	44.49	72	27.27		213	47.65	103	23.79	
≥ 5	48	18.25	7	2.65		92	20.58	11	2.54	
ER status					0.508					0.837
Positive	226	85.93	232	87.88		406	90.83	395	91.22	
Negative	37	14.07	32	12.12		41	9.17	38	8.78	
PR status					0.057					0.181
Positive	184	69.96	204	77.27		367	82.10	340	78.52	
Negative	79	30.04	60	22.73		80	17.90	93	21.48	
Histological grade					0.002					0.001
I	16	6.08	32	12.12		23	5.15	30	6.93	
II	116	44.11	131	49.62		229	51.23	270	62.36	
III	126	47.91	90	34.09		184	41.16	123	28.41	
Unknown	5	1.90	11	4.17		11	2.46	10	2.31	
Stage					<0.001					<0.001
I	0	–	196	74.24		0	–	328	75.75	
II	146	55.51	65	24.62		246	55.03	97	22.40	
III	110	41.83	3	1.14		190	42.51	4	0.92	
IV	5	1.90	0	–		7	1.57	1	0.23	
Unknown	2	0.76	0	–		4	0.89	3	0.69	

*LN* lymph node, *ER* estrogen receptor, *PR* progesterone receptor; *p* value is derived from the univariable association analyses between each of the clinicopathologic variables and LN status

Given the purpose to achieve flexible utility of available clinicopathologic features to preoperative predict the metastatic lymph nodes, factors including age at diagnosis, ethnicity, gender, laterality, tumor size, ER and PR status were further analyzed in binary logistic regression analysis. The results conveyed that age at diagnosis, ER status and tumor size were independent factors involving the positivity of axillary lymph nodes and were then incorporated into the development of the nomogram as indicated in Table 3.

**Table 3:** Logistic regression analysis to identify risk factors in IMPC of the breast in training cohort

Variables	β	Odds Ratio (95% CI)	*p*
Age at diagnosis	−1.389	0.249 (0.097 to 0.638)	0.004
Ethnicity	−0.194	0.823 (0.457 to 1.483)	0.517
Gender	−2.552	0.078 (0.003 to 1.863)	0.115
Laterality	−0.414	0.661 (0.330 to 1.323)	0.242
Tumor size(cm)	−3.462	0.031 (0.009 to 0.108)	<0.001
ER status	1.659	5.254 (0.392 to 19.834)	0.014
PR status	−0.435	0.647 (0.237 to 1.768)	0.396
Histological grade	−0.414	0.982 (0.565 to 1.707)	0.949
Stage	8.131	3396.518 (604.285 to 19,090.876)	<0.001

Β is the regression coefficient. *ER* estrogen receptor, *PR* progesterone receptor

### Nomogram development

**Fig. 1:**
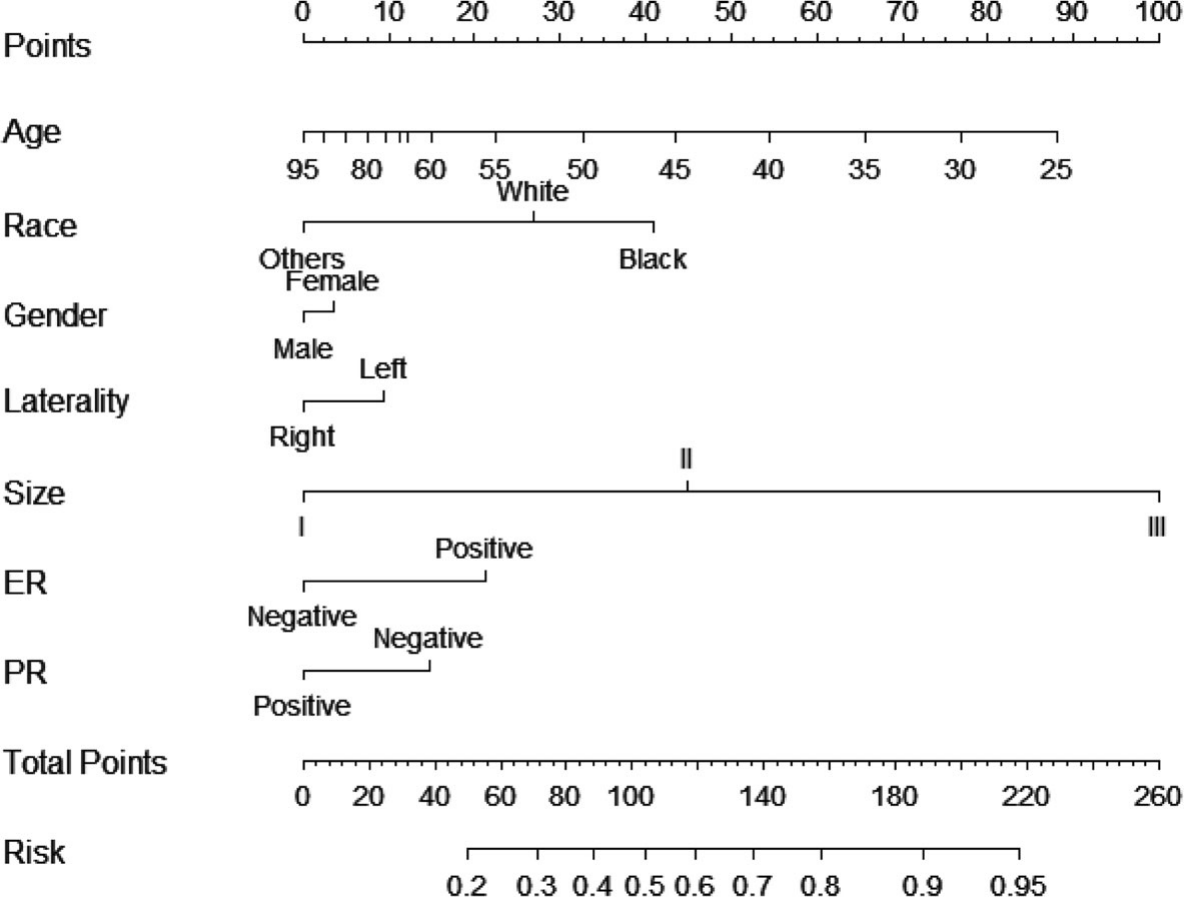
Nomogram predicting lymph node involvement in patients with invasive micropapillary carcinoma of the breast. The top row shows the point assignment for each variable. Rows 2–8 indicate the variables included in the nomogram. For an individual patient, each variable is assigned a point value based on the tumor characteristics. The points assigned to each of the seven variables are summed, and the total points indicated in row 9. The bottom row shows the probability of the patient having lymph node metastasis. ER, estrogen receptor; PR, progesterone receptor

A nomogram to predict preoperative axillary lymph nodes positivity was developed in primary cohort. A logistic regression analysis identified age at diagnosis, tumor size and ER status were risk factors (Table 3). The model that incorporated the above independent predictors in conjunction with parameters previously shown to be associated with axillary lymph node metastasis, including gender, laterality, ethics and PR status [16, 17] was developed and presented as the final nomogram (Fig. 1).

### Internal and external validation of the model

**Fig. 2:**
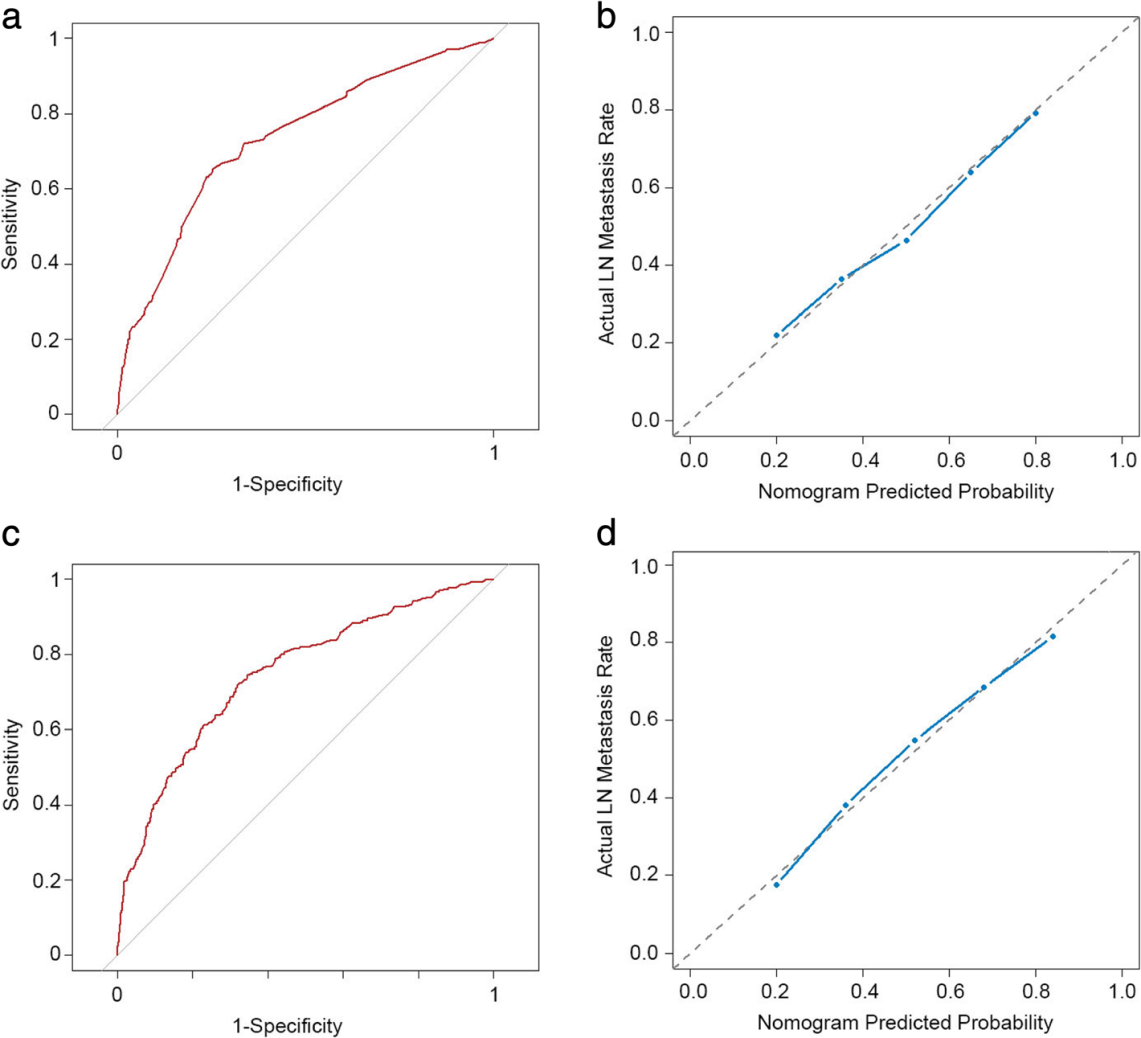
Discrimination and calibration of the nomogram in primary and validation cohorts. **a** and **c** Receiver operating characteristic (ROC) curve for discrimination in the training and validation sets. The area under the curve (AUC) of the nomagram was 0.735 (95% CI 0.692 to 0.777) and 0.748 (95% CI 0.701 to 0.767) respectively, demonstrating very good prediction performance. **b** and **d** Calibration plot of actual (observed) and predicted probabilities for the nomogram in the training and validation sets. The x-axis represents the nomogram predicted probabilities as measured by logistic regression analysis, and the y-axis the actual probabilities. Vertical lines indicate the frequency distribution of predicted probabilities. The dotted line indicates the ideal reference line where predicted probabilities match the observed probabilities. The calibration curve indicated excellent calibration of the nomogram

The nomogram was internally verified using the bootstrap validation method. The nomogram demonstrated good accuracy for predicting positive axillary lymph nodes, with an unadjusted concordance index (C-index) of 0.735 (95% CI, 0.692 to 0.777).

(Fig. 2a) and was subjected to bootstrapping validation (1000 bootstrap resamples) to calculate a relatively corrected C-index of 0.734. Calibration curves for estimating positive axillary lymph nodes indicated there was no apparent departure from perfect fit, with good correlation between the prediction and observation in the primary cohort (Fig. 2b).

Good calibration was observed for the probability of lymph node metastasis in the validation cohort (Fig. 2d), and the C-index of the nomogram for the prediction of lymph node status was 0.748 (95% CI, 0.701 to 0.767) (Fig. 2c).

## Discussion

IMPC is a rare subtype of breast carcinoma listed by the World Health Organization histologic classification of tumors of the breast in 2003, and presumed to be more aggressive than invasive ductal carcinoma (IDC) [3]. In agreement with the previous IMPC series, the positive rate of ER ranges from 25 to 91%, 13% to 82% of PR, as well as 36% to 100% of HER2 [18–25]. In our study, the positive rate of ER, PR and HER2 is 89.48%, 77.83% and 19.43% respectively. Besides, the positivity of involving lymph nodes, histological grade, tumor size and stage is similar to that of previous researches.

Although substantial controversy existed, most previous studies had demonstrated that IMPC was associated with advanced and aggressive clinicopathologic features, and the survival outcomes of IMPC was generally accepted to be worse than those of IDC [26–28]. To make matters worse, the standard treatment strategy of IMPC is unavailable and the rather low frequency of IMPC renders it difficult to directly compare its clinical outcomes and pathologic features to IDC. According to previous investigation, there were some commons and differences between IMPC and IDC in the aspect of contributors to lymph node involvement. Although some controversy existed, hormonal receptor status and tumor size were generally accepted to account for lymph node involvement both in IMPC and IDC, which was consistent with our results. While histological type, lymphovascular invasion (LVI), number of involved sentinel lymph nodes and extranodal extension were assumed to attribute to lymph node involvement of IDC, and their effects on IMPC need to be further research [29–32]. Thus, currently almost all IMPC patients were treated according to standard IDC treatments [33].

However, previous reports of IMPC had confirmed its distinctly morphological and genetic profiles, thus established IMPC of the breast as a special entity. Most patients with IMPC presented with axillary lymph node metastasis at initial diagnosis, the reported lymphatic and lymph nodal spread ranged from 33 to 95% higher than that of IDC [2, 20, 24, 25]. However, the underlying mechanisms were poorly understood, with rare laboratory studies indicated that some regulatory molecules involving with the lymphotropism. Notably, lymph node metastasis was the most important prognosis predictor of IMPC and IDC. Some uncertain and controversial remained concerning the value of sentinel lymph node biopsy (SLNB) in IMPC setting [7].

Management strategies that avoid axillary invasive procedures are needed for lymph node negative patients. If we can predict the state of the axillary lymph nodes before sentinel lymph node biopsy, individuals who are axillary negative could avoid the unnecessary axillary operation. However, the preoperative clinical and imaging examinations of the axilla is a multifactorial event. Moreover, Paterakos et al. had suggested patients with IMPC may not benefit from SLNB [7]. Therefore, the accurate predict and appropriate dispose of axillary lymph nodes shows important clinical significance.

To some extent, to help guide axillary lymph nodes treatment decisions in this subpopulation, we adopted the large-scale population-based SEER database to develop and validate a convenient nomogram for the preoperative individualized prediction of lymph node metastasis in patients with IMPC. By univariate and multivariate analysis, age at diagnosis, ER status and tumor size were independent factors involving the positivity of axillary lymph nodes, and PR had marginal correlation with that. In consideration of previously identified factors, gender, laterality and ethics, contributed to the involvement of lymph nodes. We took these factors to construct nomogram. The nomogram incorporated preoperatively available clinical and certain pathological parameters and had some utility in clinical practice with good discrimination and calibration. With this nomogram, the patients’ axillary lymph nodes status could be accurately predicted, and the clinical practice, especially the surgery planning could be made precisely and individually.

Although the nomogram showed good accuracy for predicting axillary lymph node metastasis, there were some limitations to the data that must be considered when interpreting the results. First of all, the use of retrospective data introduced the possibility of selection bias. Moreover, the vast majority of patients in the study cohort were white, so the estimation may be less precise for patients of nonwhite race. Additionally, the duration of follow-up time in this study should be longer to meet the accurate prediction as survival of breast cancer patients had dramatically improved with the advanced recognition of the nature of this malignancy. Despite these limitations, the established nomogram showed the bootstrap-corrected C-index of 0.734 indicated a sufficient level of accuracy in which C-indices generally range from 0.6 to 0.8.

In summary, we used population-based cohort to develop a nomogram to preoperatively estimate axillary lymph node metastasis in IMPC patients. This clinically useful tool applying readily available clinicopathologic factors to estimate the propensity of positive lymph node and can further facilitate individualized clinical decision making. Of note, the extrapolation of this conclusion to other database should be cautious and the following points should be taken into account. On the one hand, the definitions or evaluation criteria of ER/PR/HER2 status are not clear in this database, and some differences can exist between time periods as indicated by ASCO/CAP and its updates, thus some discrepancies may exist between training and validation sets. On the other hand, the study cohort includes data related to post neoadjuvant chemotherapy surgical samples, and these cases may have been down staged, thus may result in undervaluation of lymph node involvement. Given that, future larger sample sizes and longer follow-up prospective studies are needed to determine the accuracy of the nomogram model and to precisely extract the subpopulations awarding more intensively treatment and surveillance.

## Conclusion

In conclusion, we found that younger age at diagnosis, larger tumor lesions, ER negative status and advanced stage were risk factors associated with lymph nodes involvement in IMPC of the breast. Incorporating the previously reported factors with the currently identified risk factors, we constructed the nomogram for preoperatively predict the axillary lymph nodes status with good discrimination and calibration both in the training and validation cohorts. This accessible nomogram will facilitate to making treatment decision individually. With this nomogram, the individual axillary lymph nodes status could be accurately predicted, thus contributing to treatment decision making. Definitely, larger cohorts and/or prospective studies are warranted to further confirm our results.

## Abbreviations

AJCC: American Joint Committee on Cancer
AUC: Area under the curve
CI: Confidential interval
C-index: Concordance index
ER: Estrogen receptor
HER2: Human epidermal growth factor receptor-2
HR: Hazard ratio
IDC: Invasive ductal carcinoma
IMPC: Invasive micropapillary carcinoma
LNM: Lymph nodal metastasis
LVI: Lymphovascular invasion
PR: Progesterone receptor
ROC: Receiver operating characteristic curve
SEER: Surveillance, Epidemiology, and End Results program
SLNB: Sentinel lymph node biopsy
